# Dietary alpha‐ketoglutarate promotes beige adipogenesis and prevents obesity in middle‐aged mice

**DOI:** 10.1111/acel.13059

**Published:** 2019-11-06

**Authors:** Qiyu Tian, Junxing Zhao, Qiyuan Yang, Bo Wang, Jeanene M. Deavila, Mei-Jun Zhu, Min Du

**Affiliations:** ^1^ Department of Animal Sciences Washington State University Pullman WA USA; ^2^ College of Animal Science and Veterinary Medicine Shanxi Agricultural University Taigu China; ^3^ Department of Molecular, Cell and Cancer Biology University of Massachusetts Medical School Worcester MA USA; ^4^ School of Food Science Washington State University Pullman WA USA

**Keywords:** adipose tissue browning, aging, alpha‐ketoglutarate, DNA demethylation, glucose tolerance, high‐fat diet

## Abstract

Aging usually involves the progressive development of certain illnesses, including diabetes and obesity. Due to incapacity to form new white adipocytes, adipose expansion in aged mice primarily depends on adipocyte hypertrophy, which induces metabolic dysfunction. On the other hand, brown adipose tissue burns fatty acids, preventing ectopic lipid accumulation and metabolic diseases. However, the capacity of brown/beige adipogenesis declines inevitably during the aging process. Previously, we reported that DNA demethylation in the *Prdm16* promoter is required for beige adipogenesis. DNA methylation is mediated by ten–eleven family proteins (TET) using alpha‐ketoglutarate (AKG) as a cofactor. Here, we demonstrated that the circulatory AKG concentration was reduced in middle‐aged mice (10‐month‐old) compared with young mice (2‐month‐old). Through AKG administration replenishing the AKG pool, aged mice were associated with the lower body weight gain and fat mass, and improved glucose tolerance after challenged with high‐fat diet (HFD). These metabolic changes are accompanied by increased expression of brown adipose genes and proteins in inguinal adipose tissue. Cold‐induced brown/beige adipogenesis was impeded in HFD mice, whereas AKG rescued the impairment of beige adipocyte functionality in middle‐aged mice. Besides, AKG administration up‐regulated *Prdm16* expression, which was correlated with an increase of DNA demethylation in the *Prdm16* promoter. In summary, AKG supplementation promotes beige adipogenesis and alleviates HFD‐induced obesity in middle‐aged mice, which is associated with enhanced DNA demethylation of the *Prdm16* gene.

## Graphical abstract

The circulatory alpha‐ketoglutarate concentration was dramatically reduced in aged mice. Alpha‐ketoglutarate administration facilitates DNA demethylation in the Prdm16 promoter, which enhances its expression and brown/beige adipogenesis and improves metabolic health of aged mice challenged with high‐fat diet.

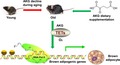

## 1 INTRODUCTION

Adipose tissue, one of the largest organs in the body, plays a central role in regulating energy homeostasis in mammalian systems (Shao et al., 2016). Excessive dietary energy leads to adipose tissue expansion, which is associated with both adipocyte hyperplasia and hypertrophy. During aging, however, the total adipocyte number declines due to progressing incapacity to replace lost adipocytes. As a result, the elders increasingly rely on existing adipocyte hypertrophy to store excess energy, resulting in hypoxia, inflammation, and metabolic dysfunction (Liang et al., 2016; Mancuso & Bouchard, 2019; Miard, Dombrowski, Carter, Boivin, & Picard, 2009).

Brown adipose tissue (BAT) increases energy expenditure via dissipating chemical energy as heat through an uncoupled respiration process (Virtanen et al., 2009). Promoting the thermogenic function of BAT and beige adipocytes reduces obesity and metabolic dysfunction. PR domain containing 16 (PRDM16) is a key transcription factor regulating brown adipogenesis and white adipocytes browning (Kajimura et al., 2008). As a key developmental gene, the *Prdm16* promoter contains CpG islands, and their DNA demethylation is required for *Prdm16* expression (Yang et al., 2016). Ten–eleven translocation family of proteins (TET) catalyze hydroxylation of 5mC to 5hmC, a key step in active DNA demethylation, which requires α‐ketoglutarate (AKG) as a cofactor (Tahiliani et al., 2009). Moreover, AKG integrates key pathways in cellular metabolism. It is an intermediate of the tricarboxylic acid cycle that is essential for the oxidation of fatty acids, amino acids, and glucose (Harrison & Pierzynowski, 2008; Xiao et al., 2016). As a precursor for the synthesis of glutamate and glutamine in multiple tissues, AKG bridges carbohydrate and nitrogen metabolism (Doucette, Schwab, Wingreen, & Rabinowitz, 2011). We found that AKG is a rate‐limiting factor controlling DNA demethylation in the *Prdm16* promoter, and its deficiency in progenitor cells profoundly attenuates brown adipogenesis (Yang et al., 2016). Recent studies showed that TET‐mediated DNA demethylation regulates the expression of proxisome‐proliferator‐activated receptor (PPAR)γ, which initiates adipocyte differentiation (Bian et al., 2018; Yoo et al., 2017). During aging, however, the cellular metabolic flux declines, which is expected to reduce the AKG concentration in nuclei and thus impede DNA demethylation and brown adipogenesis. As a small molecule, extracellular AKG can be actively absorbed and transported into cells (Burckhardt et al., 2002; Maus & Peters, 2017). Thus, we hypothesized that dietary supplementation of AKG could elevate its level in the circulation and, thus, the availability of AKG for beige adipogenesis.

To detect the effects of AKG on adipose tissue browning during aging, we fed aged mice with high‐fat diet (HFD) with or without oral supplementation of AKG. We found that HFD impaired *Prdm16* expression, BAT function, and white adipose browning. On the other hand, AKG supplementation enhanced browning of adipose tissue through AKG‐mediated demethylation in the *Prdm16* promoter.

## 2 RESULTS

### 2.1 Supplementation of AKG on body weight gain, glucose tolerance, and adipose tissue characteristics

**Figure 1:**
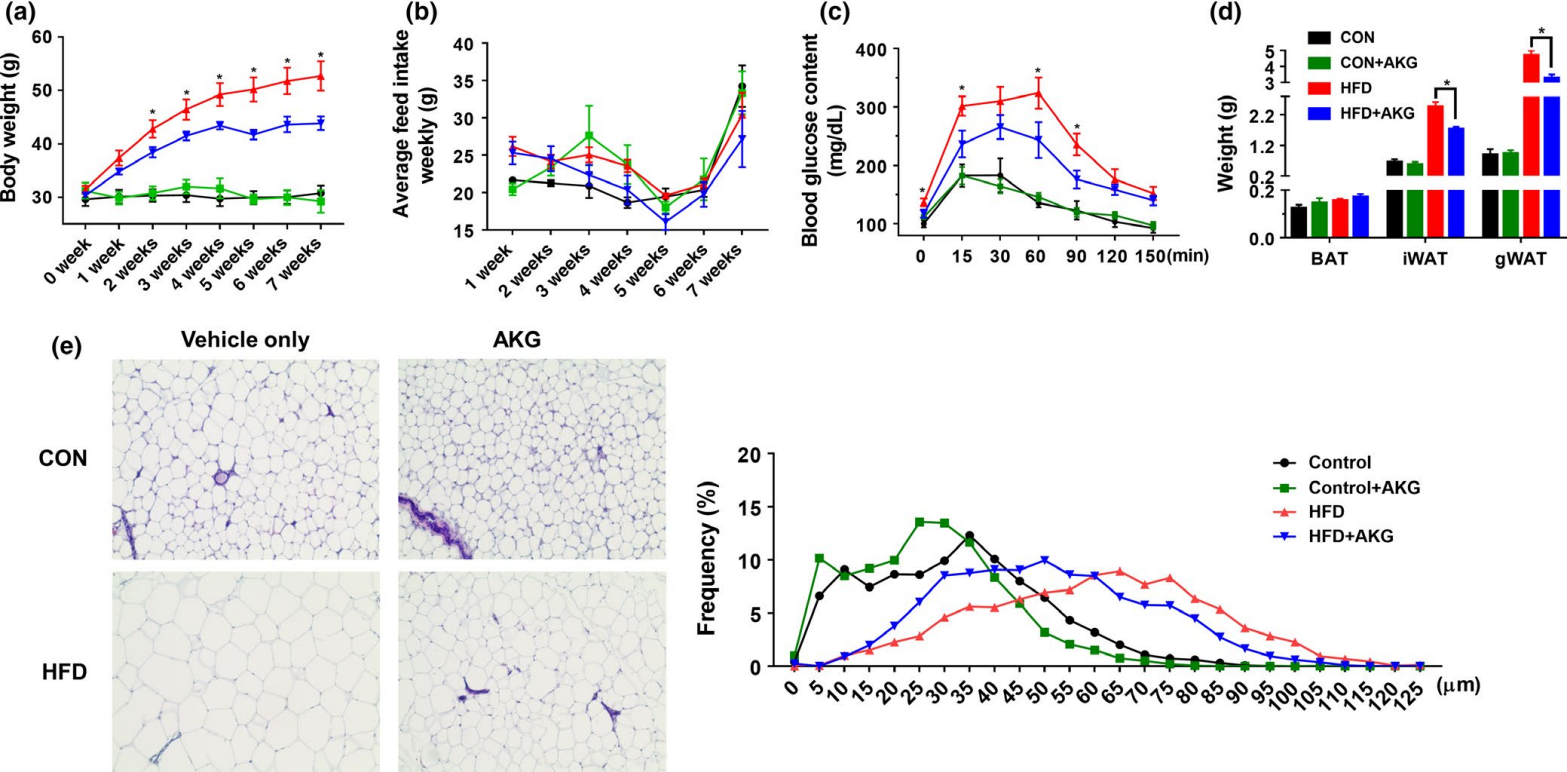
Alpha‐ketoglutarate supplementation prevents obesity and improves glucose tolerance level. Ten‐month‐old C57BL6 mice were fed either control diet or HFD and supplemented with 0 or 1% (w/v) alpha‐ketoglutarate for 2 months. (a) Body weight. (b) Food intake. (c) Glucose tolerance test. (d) Adipose tissue weight. (e) Representative images of H&E staining and adipocyte distribution of iWAT. *p < .05 (*n* = 6, mean ± *SEM*)

To determine the effect of AKG supplementation on HFD‐induced obesity during aging, AKG was added in drinking water during the whole duration of trial. Ten‐month‐old female mice were provided with either a normal chow diet or HFD (60% of calories from fat). The HFD‐fed mice exhibited increased body weight compared to the control group, whereas AKG supplementation to HFD‐fed mice significantly decreased the body weight gain from week 2. There was no difference in body weight gain between CON and CON + AKG groups (Figure 1a). AKG supplementation did not change water intake (Supporting Information Figure S1), and not affect feed intake (Figure 1b). However, the weight‐reducing effect was not observed in 2‐month‐old young mice (Supporting Information Figure S2), showing the effect of AKG on obesity prevention was specific to aged mice.

Glucose tolerance test was performed after 7 weeks of dietary treatments (Figure 1c). The basal blood glucose level of HFD group mice was higher than mice in other groups showing that oral administration of AKG improved the glucose tolerance of HFD mice. Consistently, the inguinal WAT (iWAT) and gonadal WAT (gWAT) weights of HFD group were much larger compared to the HFD + AKG group (Figure 1d). No difference in fat mass was observed between CON and CON + AKG groups. Mice in HFD group had larger lipid droplets in iWAT after two months of HFD challenge. These large adipocytes were most abundant in HFD mice, followed by HFD + AKG mice, with no difference between CON and CON + AKG mice, which was in agreement with the differences in average adipocyte diameters (Figure 1e). In summary, these data showed that AKG intake exhibits a protective effect against obesity and glucose intolerance in middle‐aged mice when challenged with HFD.

### 2.2 AKG supplementation improves metabolic rate and systemic metabolic homeostasis

**Figure 2:**
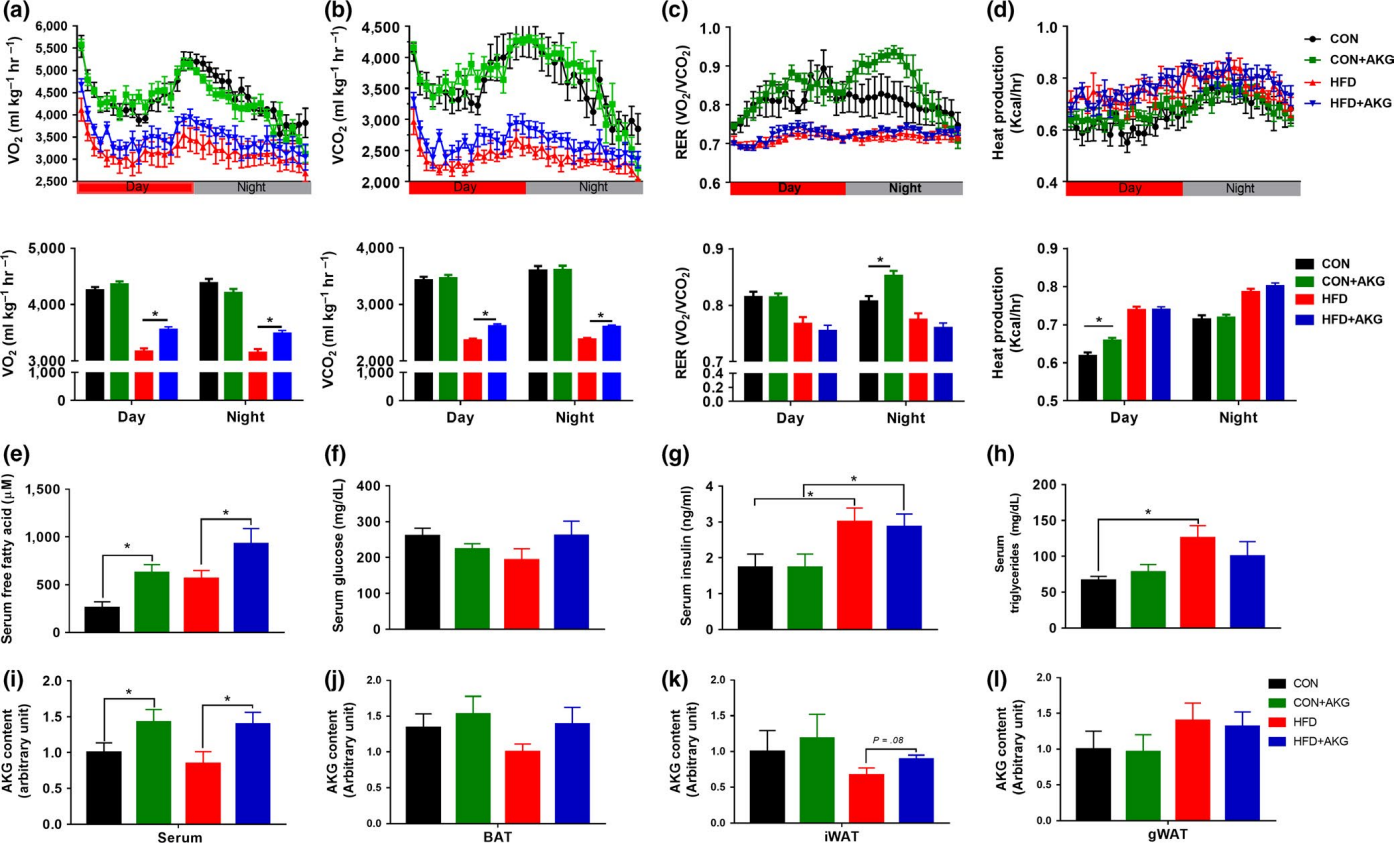
Effects of alpha‐ketoglutarate supplementation on metabolic rates and systemic metabolic homeostasis. (a) Oxygen consumption. (b) CO2 production. (c) RER. (d) Heat production. (e) Serum free fatty acids levels. (f) Serum glucose levels. (g) Serum insulin levels. (h) Serum triglycerides levels. (i) Serum AKG content. (j) BAT AKG content. (k) iWAT AKG content. (l) gWAT AKG content. *p < .05 (*n* = 6, mean ± *SEM*)

To test if AKG‐treated mice exhibited metabolic alterations, an indirect open‐circuit calorimetry system was used to measure O_2_ consumption, CO_2_ production, respiratory exchange ratio (RER), and heat production (Figure 2a–d). Mice of the HFD + AKG group showed higher O_2_ consumption and CO_2_ production during the night compared to the HFD group, whereas AKG supplementation had no effect on RER. During the night (active phase), the RER of CON mice was significantly lower than that of the CON + AKG mice, showing that CON + AKG mice preferentially utilized more carbohydrates rather than lipids as an energy source, an indicator of better metabolic flexibility. During the light cycle, CON + AKG mice displayed an increase in heat production compared to CON mice, suggesting higher basal energy expenditure.

Alpha‐ketoglutarate supplementation increased serum free fatty acid content in mice fed either control diet or HFD (Figure 2e), suggesting increased lipolysis and hypermetabolic response. HFD mice showed higher serum insulin concentration than CON mice (Figure 2g). However, no difference was observed between mice fed with/without AKG. Compared to mice fed HFD, mice fed the control diet had lower serum triglycerides content (Figure 2h). Then, we analyzed AKG contents in the circulation of young and middle‐aged mice. Interestingly, the level of blood AKG dramatically decreased in middle‐aged mice compared with young mice (Supporting Information Figure S3). Decreased AKG levels during aging were rescued by AKG supplementation, as shown by a significant increase of blood AKG in both CON and HFD‐fed animals after AKG treatment (Figure 2i). AKG levels in different adipose tissues were further measured to test whether AKG was accumulated among these adipose depots (Figure 2j–l). The AKG levels in iWAT were higher in HFD + AKG group compared to HFD group. However, the AKG levels in BAT and gWAT were not affected by AKG supplementation. In young mice, HFD group had lower level of AKG in iWAT compared with CON mice fed with/without AKG. Compared to CON mice, HFD‐fed mice had lower AKG levels in gWAT. The AKG levels in BAT, iWAT, and gWAT were not altered by AKG supplementation (Supporting Information Figure S4a–c).

### 2.3 Beige/brown adipogenesis is enhanced in mice supplemented with AKG

**Figure 3:**
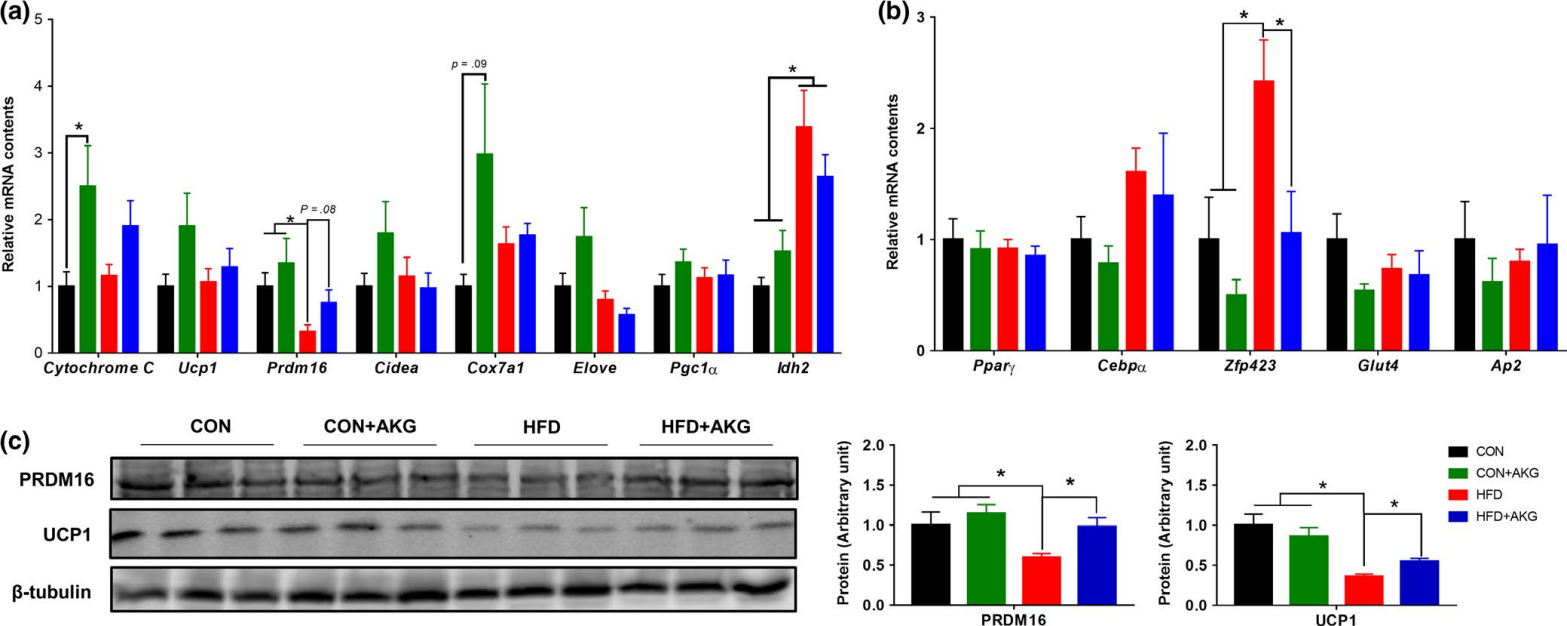
Alpha‐ketoglutarate supplementation promotes beige adipogenesis in inguinal white adipose tissue (iWAT). (a) Brown adipose gene mRNA levels in iWAT analyzed by q‐PCR. (b) White adipose gene mRNA levels in IngWAT analyzed by q‐PCR. (c) Brown adipose protein contents in iWAT analyzed by Western blot. *p < .05 (*n* = 6, mean ± *SEM*)

**Figure 4:**
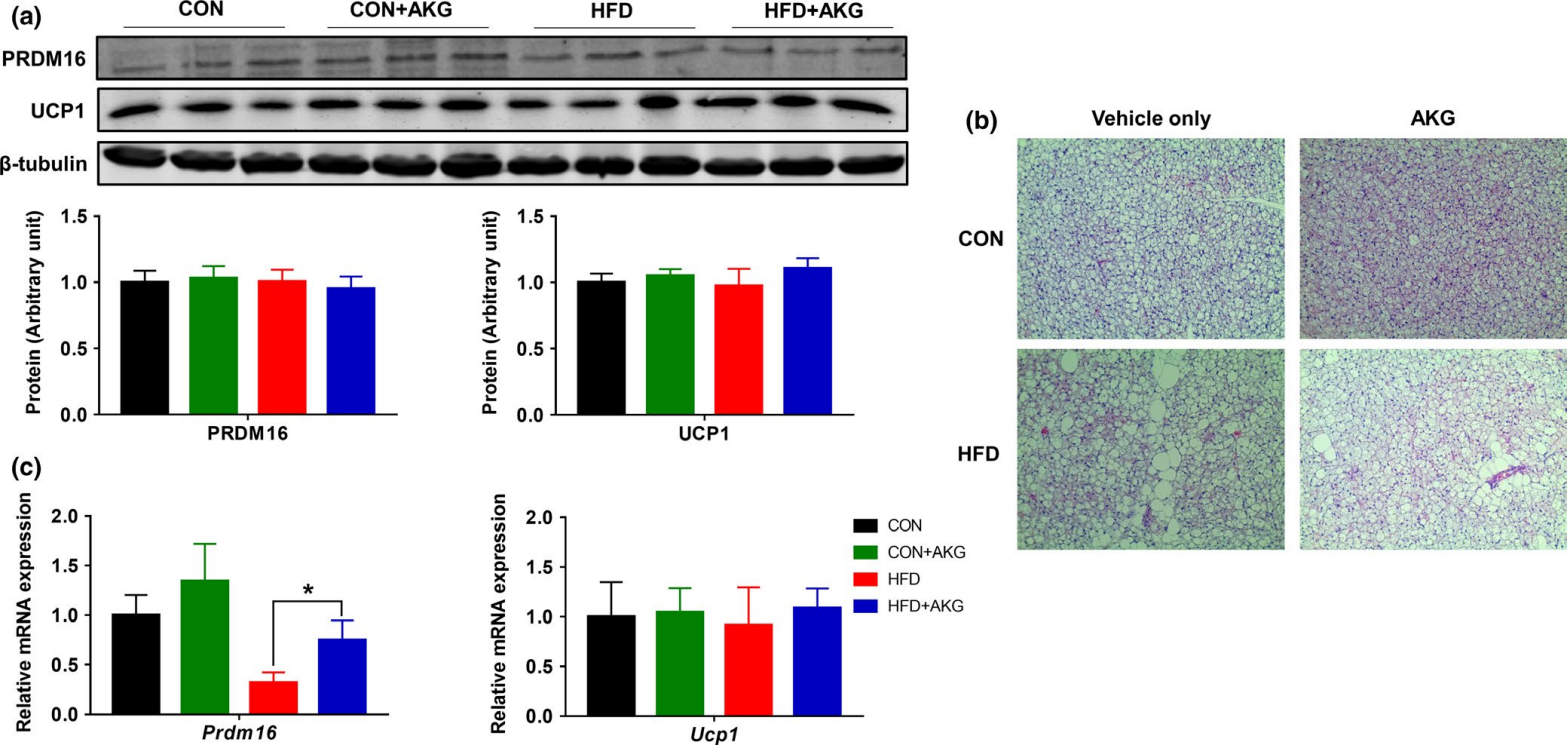
Effect of alpha‐ketoglutarate supplementation on metabolic activity of BAT. (a) PRDM16 and UCP1 contents in BAT analyzed by Western blot. (b) Representative H&E staining of BAT. (c) *Prdm16* and *Ucp1* mRNA levels in BAT analyzed by q‐PCR. *p < .05 (*n* = 6, mean ± *SEM*)

To investigate the mechanisms suppressing weight gain in HFD mice supplemented with AKG, the adipogenic‐related genes and proteins were further examined. In the iWAT, the mRNA expression of *Prdm16* was lower in HFD mice but rescued by AKG supplementation (Figure 3a). AKG administration also elevated *Cytochrome C* and *Cox7α1* expression in the CON mice. No alterations were detected for white adipogenic genes, including *Pparγ* and *Cebpα* mRNA expression, in the iWAT of aged mice (Figure 3b). However, *Zfp423* mRNA expression was highly up‐regulated in iWAT of HFD mice, suggesting increased white adipogenesis. Consistent with the changes in thermogenic gene expression, UCP1 and PRDM16 protein contents in the iWAT of HFD + AKG mice were higher than HFD mice (Figure 3c). Histological staining showed that brown adipocytes in middle‐aged mice fed with HFD had increased lipid accumulation and bigger lipid droplets (Figure 4b). In BAT, *Prdm16* mRNA expression was increased in HFD + AKG group compared with HFD group (Figure 4c). However, in gWAT (Supporting Information Figure S5), AKG supplementation did not change thermogenic gene and protein expression in HFD mice. In young mice, HFD + AKG group also had higher *Ucp1* expression in BAT than HFD group, whereas no difference was observed when treated with AKG in iWAT and gWAT (Supporting Information Figure S6a–c).

### 2.4 AKG enhances cold‐induced beige/brown adipogenesis

**Figure 5:**
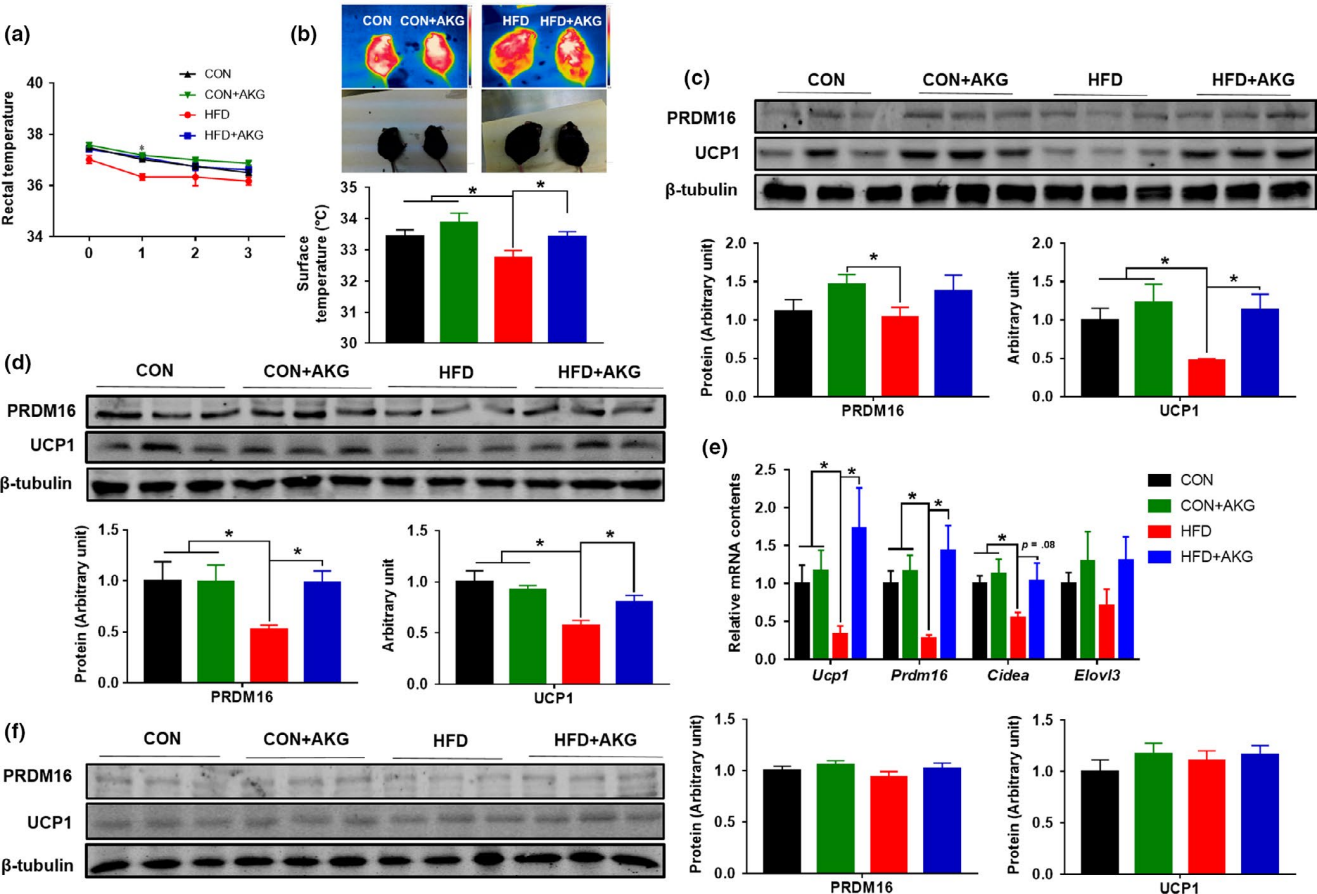
Alpha‐ketoglutarate supplementation regulates thermogenesis after cold exposure. (a) Rectal temperature. (b) Surface temperature. (c) PRDM16 and UCP1 contents in brown adipose tissue. (d) PRDM16 and UCP1 contents in iWAT. (e) *Prdm16* and *Ucp1* mRNA levels in IngWAT analyzed by q‐PCR. (f) PRDM16 and UCP1 contents in gWAT. *p < .05 (*n* = 6, mean ± *SEM*)

In response to cold exposure, inguinal white adipose tissue can generate thermogenic beige/brown adipocytes which are enriched with mitochondria and UCP1 protein (Dempersmier et al., 2015; Hui et al., 2015). To evaluate the effects of AKG on thermogenic activity, we measured the rectal temperature for 3 days during cold exposure (4°C). We found that HFD mice had a lower rectal temperature, which was recovered due to AKG supplementation. (Figure 5a). In agreement, a higher surface temperature was detected in HFD + AKG compared to HFD mice after cold exposure (Figure 5b). Consistently, the expression of both UCP1 and PRDM16 was up‐regulated in BAT of HFD + AKG mice (Figure 5c). In iWAT, AKG supplementation rescued the decline of *Ucp1* and *Prdm16* expression in both gene and protein levels of HFD mice after cold exposure (Figure 5d,e). However, the beneficial effect of AKG administration in thermogenesis was not observed in gWAT, likely due to few numbers of beige adipocytes in this fat depot (Figure 5f). The UCP1 levels in iWAT and gWAT were also confirmed by ELISA kit (Supporting Information Figure S7a,b). After cold exposure, AKG levels in iWAT were elevated in HFD + AKG group compared with HFD group. In BAT, mice fed the control diet had higher AKG levels, and HFD + AKG mice showed higher AKG levels than HFD mice as well (Supporting Information Figure S7c–e).

### 2.5 Dietary supplementation of AKG induces DNA demethylation in promoters of key beige/brown adipogenesis genes during cold challenge

**Figure 6:**
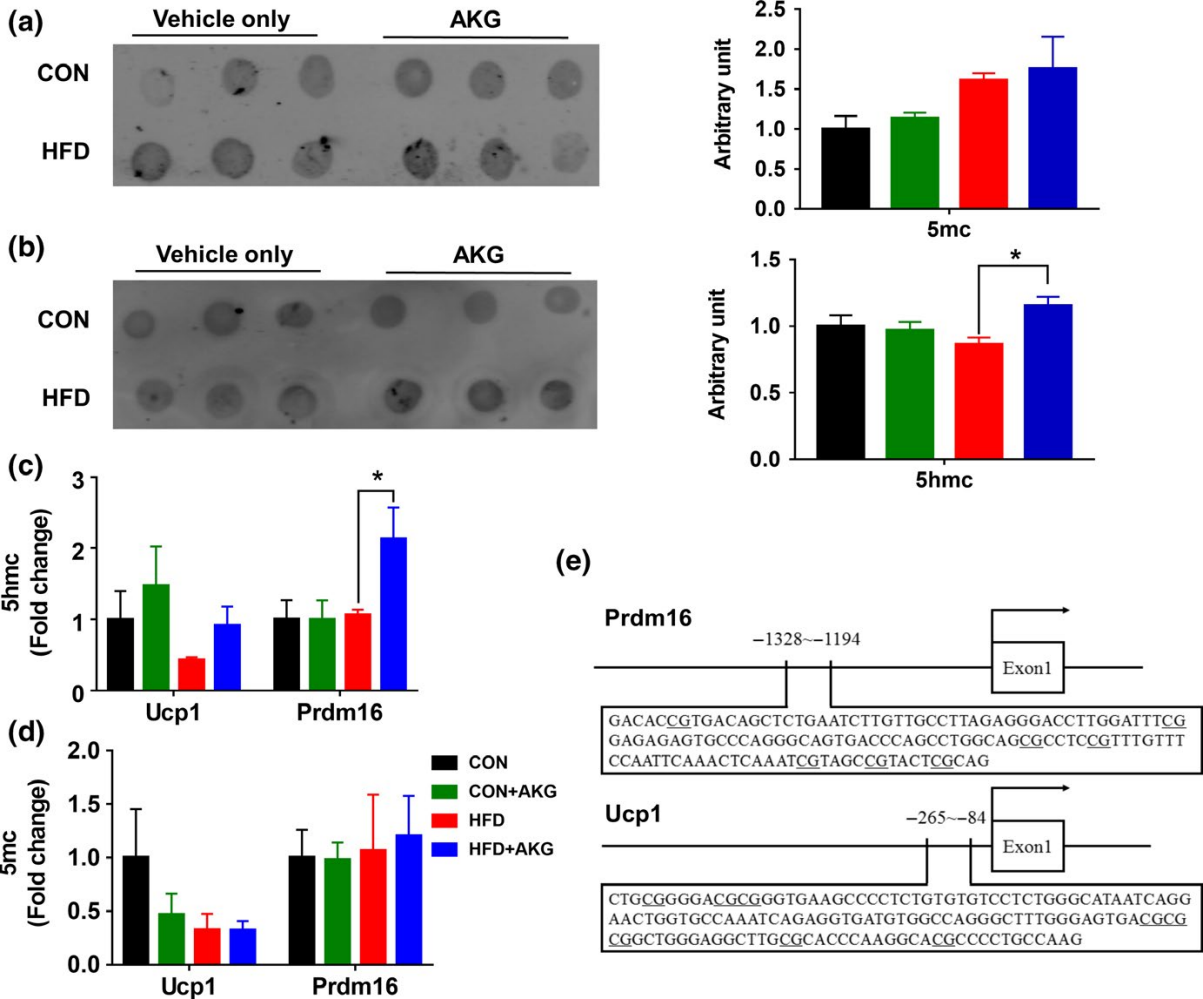
Global DNA methylation level, DNA methylation of the *Ucp1* and *Prdm16* promoters after cold exposure. (a) Dot blot analysis of 5‐mC in DNA extracted from iWAT after cold exposure. (b) Dot blot analysis of 5‐hmC in DNA extracted from iWAT after cold exposure. (c) Enrichment of 5mC at promoter regions relative to input normalized by the positive control in iWAT after cold exposure. (d) Enrichment of 5hmC at promoter regions relative to input normalized by the positive control in iWAT after cold exposure. (e) Schematic diagram showing the promoter sequences of mice *Ucp1* and *Prdm16* genes. *p < .05 (*n* = 6, mean ± *SEM*)

We analyzed *TET* expression levels in young mice and middle‐aged mice. Young mice had higher *TET1* and *TET2* levels in BAT than middle‐aged mice (Supporting Information Figure S8a). As AKG is an important mediator of active DNA demethylation process, the global DNA methylation levels in iWAT were analyzed by dot blot. As expected, the level of 5hmC, an intermediate of DNA demethylation, was increased in HFD + AKG compared to HFD mice (Figure 6a,b). Previously, we found that AKG mediates active DNA demethylation in the *Prdm16* promoter (Yang et al., 2016). Thus, the enrichment of 5mC and 5hmC in the promoters of *Ucp1* and *Prdm16* genes were further determined using the MeDIP analysis where are shown schematically in Figure 6e. AKG supplementation did not alter 5mC content in genes associated with beige/brown adipogenesis among different groups, which could be explained by the presence of abundant nonadipogenic cells in iWAT, masking the changes of 5mC in adipogenic cells. On the other hand, an important indicator, the presence of 5hmC in the *Prdm16* promoter which is only present in brown/beige adipogenic cells was elevated in HFD + AKG compared to HFD mice (Figure 6c). The *Ucp1* expression had also been reported to be regulated by DNA methylation (Shore, Karamitri, Kemp, Speakman, & Lomax, 2010). However, no change in DNA methylation of the *Ucp1* promoter was detected. Our results suggested that dietary AKG up‐regulated *Prdm16* expression and beige/brown adipogenesis partially through facilitating active DNA demethylation. DNA methylation of white adipogenic marker *Zfp423* at the promoter region was further analyzed. The 5mc and 5hmc levels of *Zfp423* were not changed by AKG supplementation (Supporting Information Figure S9a,b).

## 3 DISCUSSION

Obesity is caused by an imbalance between energy consumption and expenditure. Adipose tissue can be separated into WAT and BAT. White adipose tissue stores excessive energy through adipocyte hypertrophy and hyperplasia (Rosen & Spiegelman, 2014). On the other hand, BAT burns fatty acids and glucose to generate heat (Virtanen et al., 2009). Additionally, beige adipocytes have recently been identified in WAT which is inducible (Harms & Seale, 2013). During aging, brown‐to‐white transitioning was observed in subcutaneous WAT (Rogers, Landa, Park, & Smith, 2012). As noted before, aging also leads to a decline of human BAT either in mass and activity (Cypess et al., 2009; Graja, Gohlke, & Schulz, 2018; Pfannenberg et al., 2010). From our recent study, we found that brown adipogenesis was modulated by DNA demethylation of brown adipogenic genes (Yang et al., 2016). AKG is an essential cofactor for TET‐mediated DNA demethylation. The TET family of proteins, TET1, TET2, and TET3, catalyzes hydroxylation of 5mC to 5hmC (Tahiliani et al., 2009), a key step in active DNA demethylation which is dependent on the presence of AKG (Ito et al., 2010; Tahiliani et al., 2009; Wang et al., 2013).

During aging, the cellular metabolic flux declines and the AKG concentration decreases dramatically (Harrison & Pierzynowski, 2008), consistent with observations of the present study. The limited availability of AKG in aged mice is postulated to impede DNA demethylation, which is required for the differentiation of adipogenic progenitor cells (Yang et al., 2016). Indeed, aberrant methylation of CpG islands in nonimprinted autosomal genes gradually increases during aging (Christensen et al., 2009; Jones & Laird, 1999; Richardson, 2003). Obesity accelerates epigenetic aging, which is worsened in middle‐aged subjects (Horvath et al., 2014; Nevalainen et al., 2017). Here, we demonstrated that AKG functions not just as an intermediate in metabolic pathways but also as an epigenetic regulator during the aging process. AKG administration replenished the AKG pool which becomes shallow due to aging and enhanced brown/beige adipogenesis through facilitating DNA demethylation.

Both white and beige adipocytes are derived from platelet‐derived growth factor receptor α (PGDFRα) positive cells (Catalano & Hauguel‐De Mouzon, 2011; Joe et al., 2010; Uezumi, Fukada, Yamamoto, Takeda, & Tsuchida, 2010; Uezumi et al., 2011). PGDFRα positive cells can differentiate into both brown and white adipocytes upon activation of β3‐adrenergic signaling or cold exposure (Lee, Petkova, Mottillo, & Granneman, 2012; Ramseyer & Granneman, 2016), which involves active DNA demethylation in the *Prdm16* promoter (Yang et al., 2016). Consistent with the limited availability of AKG in aged mice, under cold stimulus, DNA demethylation in the *Prdm16* promoter was hampered in aged mice. Because AKG is absorbable by cells (Maus & Peters, 2017), we supplemented middle‐aged mice with AKG and found that dietary supplementation of AKG could elevate their circulatory level and the availability of AKG for beige adipogenesis. We found AKG was elevated in BAT and iWAT after cold exposure, which acted as a cofactor facilitating the DNA demethylation of brown adipogenesis genes. As a result, thermogenesis‐associated protein UCP1 was enhanced in iWAT by AKG intake. Cold exposure is a standard method to induce the browning of WAT and increase thermogenesis in BAT (Bhatt, Dhillo, & Salem, 2017; Dempersmier et al., 2015). Consistently, the UCP1 expression and thermogenic function of middle‐aged mice supplemented with AKG were further enhanced by cold exposure. The supplementation of AKG did not alter 5mC content in genes associated with beige/brown adipogenesis among different groups, which could be due to the presence of abundant DNA in cells without brown/beige adipogenic capacity. In agreement, AKG administration in aged mice enhanced 5hmC, showing that DNA demethylation in *Prdm16* promoter and brown/beige adipogenesis were enhanced by AKG. The body weight gain of HFD mice supplemented with AKG was lower than that of HFD mice. Moreover, AKG supplementation improved glucose tolerance in mice challenged with HFD. To date, no previous study has assessed the effect of AKG supplementation on adipocyte browning and metabolic rate in HFD‐fed mice.

Our data showed that the beneficial effect of AKG on brown/beige adipogenesis was limited to BAT and iWAT, not in gWAT, which could be due to the difference in cellularity among these fat depots. The capacity of the adipose tissue to expand relies on adipocyte hyperplasia and hypertrophy, which highly depends on the presence of adipogenic progenitor cells (Baptista, Silva, & Borojevic, 2015). However, the density of progenitor cells differ among fat depots, and their density in much higher in subcutaneous compared to visceral fat (Ong et al., 2014), which commonly locate in the external layer of blood vessel wall (Silva et al., 2017; Zimmerlin et al., 2010). The scarcity of progenitor cells in the visceral fat renders its preferential growth by hypertrophy, while subcutaneous fat grows preferentially by hyperplasia (Joe, Yi, Even, Vogl, & Rossi, 2009). Such a difference in cellularity might explain the lack of response to AKG supplementation in the gWAT of aged mice.

In this study, we used 1% of AKG in drinking water for dietary supplementation, which is within the dietary intake range of AKG in humans. As a major intermediate of the tricarboxylic acid cycle and a derivative of dietary glutamine and glutamate, major amino acids, the AKG contents in foods are not scarce. Interestingly, AKG is also a product of gut microbiota (Otto, Yovkova, & Barth, 2011), and the metabolic impacts of AKG derived from microbiota and their interaction with food components need to be further explored.

In summary, our data showed that the circulatory AKG concentration was reduced in middle‐aged mice, which limits the plasticity of adipogenic progenitor cells undergoing beige adipogenesis. Dietary supplementation of AKG increased intracellular levels of AKG and enhanced beige adipogenesis, which improved metabolic health of aged mice challenged with HFD. Because active DNA demethylation is not only limited to brown/beige adipogenesis but also presents in the differentiation of progenitor cells in other tissues, the dietary AKG intervention might have preventive effects in the senescence of other tissues as well, which warrant further studies.

## 4 EXPERIMENTAL PROCEDURES

### 4.1 Animals and experimental design

All animal use protocols were reviewed and approved by the Institutional Animal Use and Care Committee (IAUCC) at Washington State University. Briefly, twenty‐four female C57BL/6 mice at 10 month of age and twenty‐four female C57BL/6 mice at 2 months of age were used in this study (*n* = 6). Mice were assigned to four groups, fed either a control diet (10% energy from fat, D12450H; Research Diets) or a high‐fat diet (HFD) (60% energy from fat, D12492; Research Diets), and receiving either 0 or 1% AKG (Alfa Aesar) in drinking water for 8 weeks. Weight gain and food intake were monitored weekly. At the end of the experiment, mice were euthanized by carbon dioxide inhalation and cervical dislocation. Blood samples were collected by cardiac puncture. The interscapular BAT, iWAT, and gWAT were rapidly isolated and weighed.

### 4.2 Glucose tolerance test (GTT)

One week before euthanization, following a 12‐hr fasting, mice were injected with D‐glucose (1 g/kg). Blood samples were collected from the tail vein at 0, 15, 30, 60, 90, and 120 min after glucose administration, and glucose concentrations were measured using a glucometer (Bayer Contour).

### 4.3 Cold exposure and tolerance test

Mice were individually housed in precooled cages and exposed to cold temperature (4°C) for 3 days with free access to food and water. The rectal temperature of mice was measured daily in the rectum at 2.5 cm in depth using a digital thermometer (Thermalert TH‐5; Physitemp). After cold exposure, mice were euthanized.

### 4.4 Metabolic chamber measurement

Metabolic rates of mice were analyzed using the metabolic chamber as previously described (Zou et al., 2017). Each mouse was housed individually and oxygen consumption (VO_2_), carbon dioxide production (VCO_2_), respiratory exchange ratio (RER), and heat production were recorded simultaneously using an Oxymax indirect open‐circuit calorimetry system (Columbus Instruments) in a constant environmental temperature. Data were collected continuously for 24 hr with 12‐hr light and 12‐hr dark. Measurements were taken every 30 min.

### 4.5 Histological analysis

Adipose tissues were fixed for 24 hr at room temperature in PBS containing 4% paraformaldehyde and then embedded in paraffin. Tissue sections (5 μm) were deparaffinized, rehydrated, and used for hematoxylin and eosin (H&E) staining. At least four images per section and four sections from each individual mouse were analyzed. Adipocyte diameters were measured by ImageJ 6.0 (Media Cybernetics, Inc.).

### 4.6 Determination of blood metabolite profile

Serum insulin concentration was measured using a Mouse Ultrasensitive Insulin ELISA Kit (no. 80‐INSMSU‐E10; ALPCO Diagnostics). Serum triglyceride and free fatty acid concentrations were measured by a Triglyceride Colorimetric Assay Kit from Cayman (no. 10010303) and an EnzyChrom Free Fatty Acid Assay Kit (BioAssay Systems), respectively.

### 4.7 GC/MS measurements for AKG

GC/MS analysis was performed using an Agilent 6890 GC equipped with a 30‐m Rtx‐5 capillary column connected to an Agilent 5975B MS. For metabolites analysis, the following heating cycle was used for the GC oven: 100°C for three min, followed by a temperature increase at 5°C/min to 300°C for a total run time of 48 min. Data were acquired in scan mode. The abundance of relative metabolites was calculated from the integrated signal of all potentially labeled ions for each metabolite fragment (Showalter et al., 2017).

### 4.8 Quantitative real‐time PCR

Total RNA was extracted from iWAT, gWAT, and BAT using TRIzol reagent (Sigma) according to the manufacturer's instructions. Total RNA (500) ng was reverse‐transcribed to cDNA using an iScript^TM^ cDNA Synthesis Kit (Bio‐Rad). Real‐time quantitative PCR was carried out using the CFX RT‐PCR detection system (Bio‐Rad) as described previously using 18S rRNA as a reference gene (Wang et al., 2015). The relative mRNA expression was determined using the method of 2^‐ΔΔCT^ (Livak & Schmittgen, 2001). The primer sequences are listed in Supporting Information Table S1.

### 4.9 UCP1 quantification

WAT UCP1 concentration was measured using Mouse Mitochondrial brown fat uncoupling protein 1 (UCP1) ELISA Kit (KTE70038; Abbkine).

### 4.10 Immunoblotting analysis

Immunoblotting analysis was conducted as previously described using an Odyssey Infrared Image System (LI‐COR Biosciences (Zou et al., 2017)). Antibodies against β‐tubulin (no. 2,146) were purchased from Cell Signaling and were diluted 1:1,000. UCP1 polyclonal antibody (no. PA1‐24894) and PR domain containing 16 polyclonal antibody (PRDM16; no. PA5‐20872) were purchased from Thermo Scientific and were diluted 1:1,000. Band density was quantified and then normalized to β‐tubulin content, because the levels of β‐tubulin did not differ between experimental groups.

### 4.11 Methylated DNA immunoprecipitation (MeDIP) analysis

High‐quality genomic DNA was isolated from iWAT and sonicated to fragments with the size of approximately 500 bp. An aliquot of fragmented DNA (2 μg) was heat‐denatured to produce single‐stranded DNA, and a portion of the denatured DNA was stored as input DNA. Mouse monoclonal antibodies against 5‐methyl cytosine or 5‐hydroxymethyl cytosine from Zymo Research were used to immunoprecipitate DNA fragments containing methylated or hydroxymethyl cytosine. The complexes were captured using chip‐grade magnetic beads (Cell Signaling Technology). The beads were washed to eliminate nonspecific binding and resuspended in 250 μl digestion buffer containing proteinase K. Finally, the immunoprecipitated DNA was purified by Tris‐saturated phenol, chloroform, and isoamyl alcohol (25:24:1), and a small aliquot and control input DNA were used to amplify DNA sequence of the proximal promoters of *Ucp1*, *Prdm16,* and *Zfp423* genes by real‐time PCR with primers listed in Supporting Information Table S2. Data were normalized against the input and presented as the fold change relative to the average value of the control group.

### 4.12 Statistical analysis

Data are presented as means ± *SEM*. Each animal was considered as the experimental unit. The general linear model and Student–Newman–Keuls (SNK) multiple test (SAS Institute Inc.) were used to analyze data and to determine the significance of differences among means of different treatments. Young mice and middle‐aged mice data were analyzed using 2‐tailed Student's *t* test. A value of *p* < .05 was considered to be statistically significant.

## CONFLICT OF INTEREST

None declared.

## AUTHOR’S CONTRIBUTION

QT, JZ and MD were responsible for conception and design of the study; QT and JZ performed the experiments and contributed to statistical analysis; QY, BW, JMD, M‐JZ and MD contributed to critical revision of the manuscript; QT and MD finalized the text.

## Data Availability

All data generated or analyzed during this study are included in this article.
