# Supplementary material for: Dietary alpha‐ketoglutarate promotes beige adipogenesis and prevents obesity in middle‐aged mice
Source: Aging Cell. 2019 Nov 6;19(1):e13059. doi: 10.1111/acel.13059 (PMC6974731; doi:10.1111/acel.13059)
Supplement: Supplementary file 1 [file ACEL-19-e13059-s001.pdf]

Supplementary Information File:

Dietary alpha-ketoglutarate promotes beige adipogenesis and prevents obesity in middle-aged mice

Supplementary Table S1. Primer sequences used for real-time quantitative PCR

Gene	Forward (5′-3′)	Reverse (5′-3′)
**18s**	GTAACCCGTTGAACCCCATT	CCATCCAATCGGTAGTAGCG
**Cytochrome C**	TTTCAGGCTTCACCCTAGATGA	GAAGAATGTTATGTCCTACGAATATG
**Ucp1**	ACTGCCACACCTCCAGTCATT	CTTTGCCTCACTCAGGATTGG
**Prdm16**	CAGCACGGTGAAGCCATTC	GCGTGCATCCGCTTGTG
**Pgc1α**	CCCTGCCATTGTTAAGACC	TGCTGCTGTTCCTGTTTTC
**Cidea**	ATCACAACTGGCCTGGTTACG	TACTACCCGGTGTCCATTTCT
**Elovl3**	GATGGTTCTGGGCACCATCTT	CGTTGTTGTGTGGCATCCTT
**Cox7a1**	CAGCGTCATGGTCAGTCTGT	AGAAAACCGTGTGGCAGAGA
**Idh2**	CTAGAGTCCCCACCGCAC	ATCTCGTCACCGTCCATCTC
**Pparγ**	AGCTCCAAGAATACCAAAGTGCGAT	AGGTTCTTCATGAGGCCTGTTGTAGA
**Zfp423**	GTCACCAGTGCCCAGGAAGAAGAC	AACATCTGGTTGCACAGTTTACACTCAT
**Cebpα**	ATGAGAGAAGGAGGGGAGCAGG	AGGTGGGAGAGGCGTGGAACTA
**Glut4**	CTCTCAGGCATCAATGCTGTTTTCTA	CGAGACCAACGTGAAGACCGTATT
**aP2**	CGACAGGAAGGTGAAGAGCATCATA	CATAAACTCTTGTGGAAGTCACGCCT
**TET1**	CACCCTGTGACTGTGATGGAGGTA	ACTATCTTCTCAATCCGGATTGCCTT
**TET2**	AAGGATGCAATCCAGACAAAGATGAA	TTTAGCAATAGGACATCCCTGAGAGCT

Supplementary Table S2. MeDIP primer sequences for *Prdm16, Ucp1* and *Zfp423*

Gene	Forward (5′-3′)	Reverse (5′-3′)
**Medip- Prdm16**	GACACCGTGACAGCTCTGAA	CTGCGAGTACGGCTACGATT
**Medip-Ucp1**	GAGTGACAAAAGGCACCACG	GAGTGACAAAAGGCACCACG
**Medip-Zfp423**	GCACGGGCCTGTTATCTGT	AGGATGTGAGGAGCGGAGT

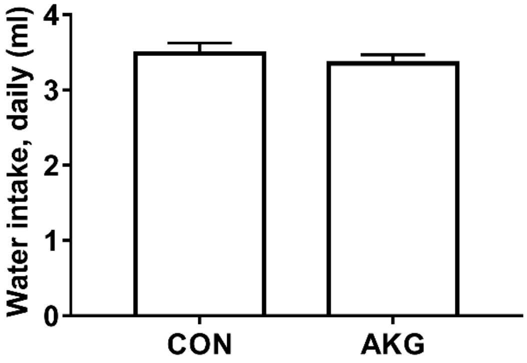

Supplementary Figure 1. Daily water intake of middle-aged mice supplemented with/without AKG. * $p < 0.05$ (n = 6, mean ± SEM).

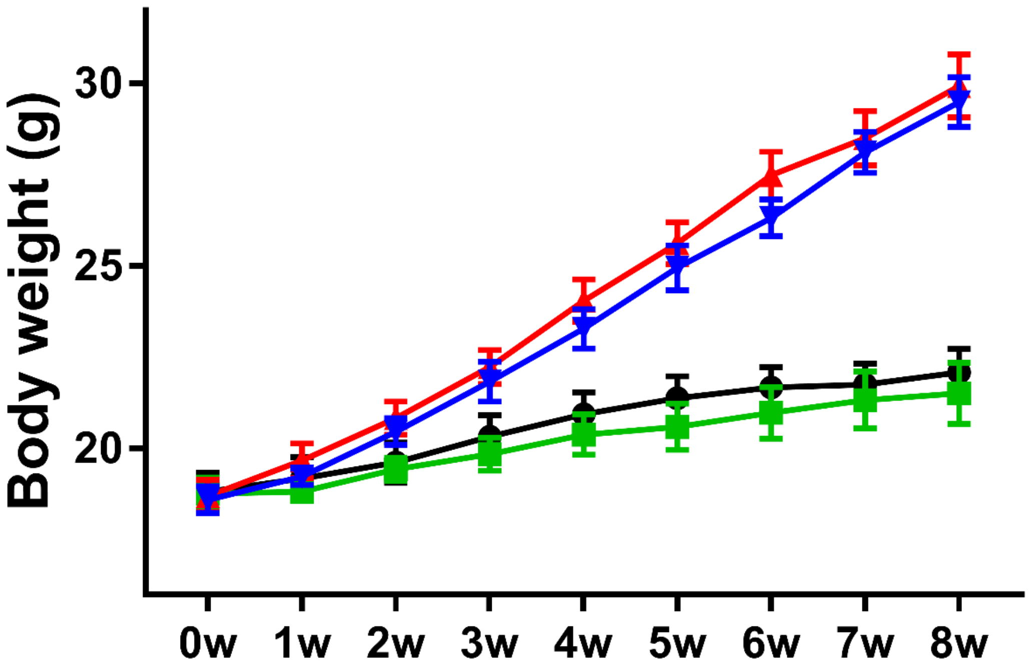

Supplementary Figure 2. Two-month-old C57BL6 mice were fed either control diet or HFD and supplemented with 0 or 1% (w/v) alpha-ketoglutarate for 2 mo. Body weight change during the treatment period. * $p < 0.05$ (n = 6, mean ± SEM).

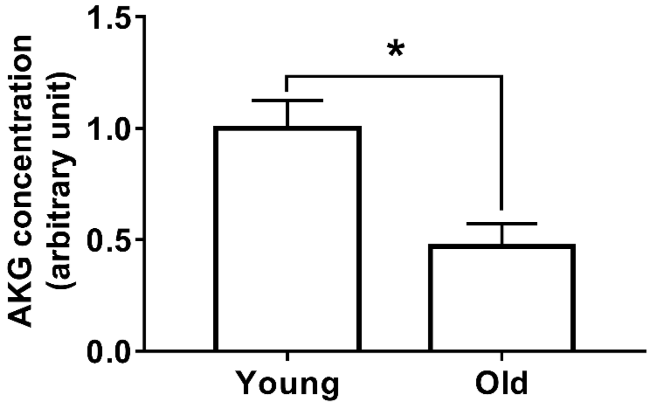

Supplementary Figure 3. Serum AKG content decreased in middle-aged mice. * $p < 0.05$ (n = 6, mean ± SEM).

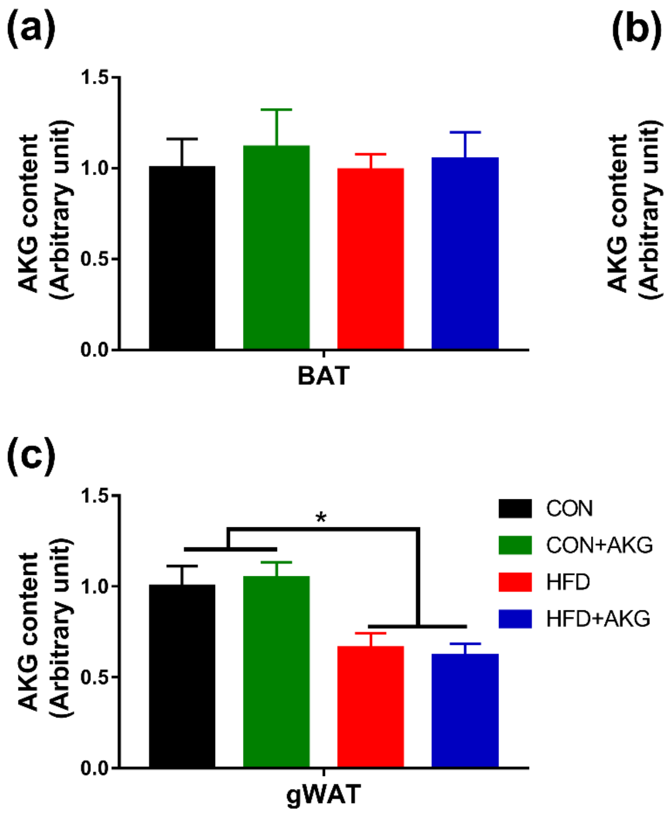

Supplementary Figure 4. AKG levels in BAT and WAT in young mice (2-month-old). (a) AKG concentration in BAT. (b) AKG concentration in iWAT. (c) AKG concentration in gWAT. * $p < 0.05$ (n = 6, mean ± SEM).

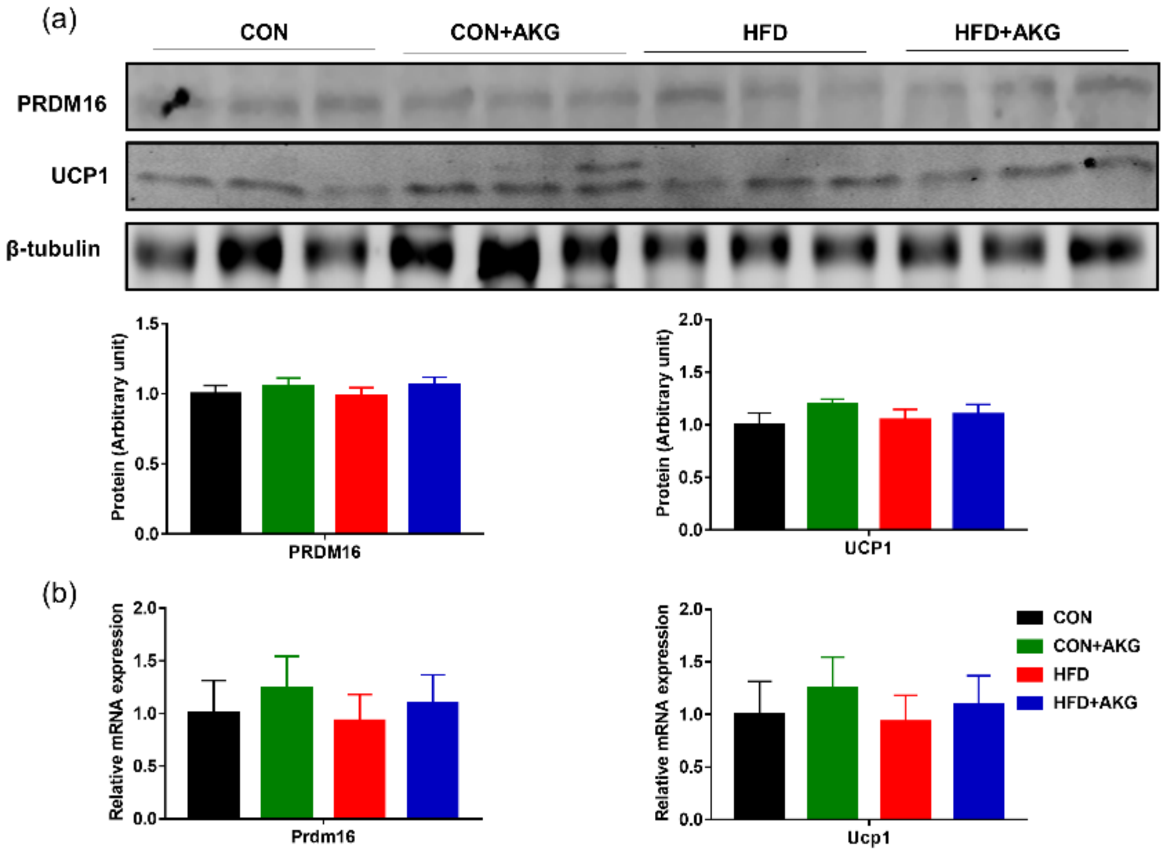

Supplemental Figure 5. Effect of alpha-ketoglutarate supplementation on metabolic activity of gWAT. (a) Protein contents of PRMD16 and UCP1 in BAT analyzed by western. (b) Brown adipose gene mRNA levels in BAT analyzed by q-PCR. * $p < 0.05$ (n = 6, mean ± SEM).

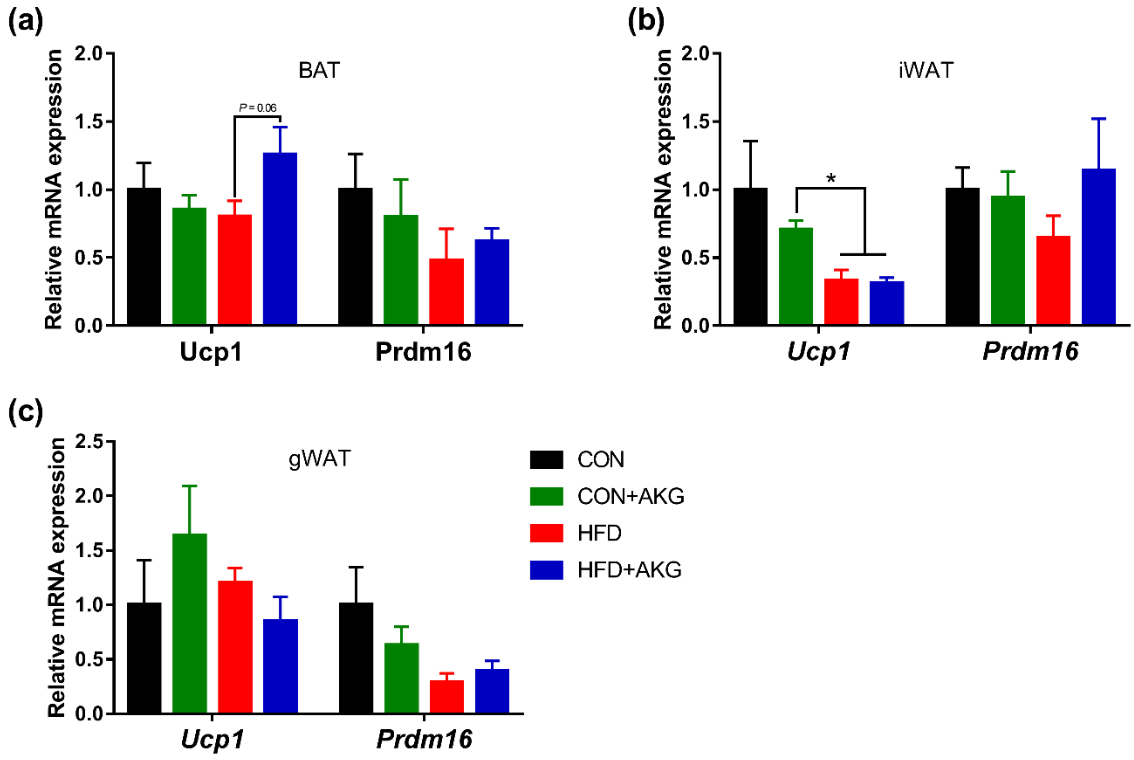

Supplementary Figure 6. Brown adipose gene mRNA levels in young mice. (a) Brown adipose gene mRNA levels in BAT analyzed by q-PCR. (b) Brown adipose gene mRNA levels in iWAT analyzed by q-PCR. (c) Brown adipose gene mRNA levels in gWAT analyzed by q-PCR. *p < 0.05 (n = 6, mean ± SEM).

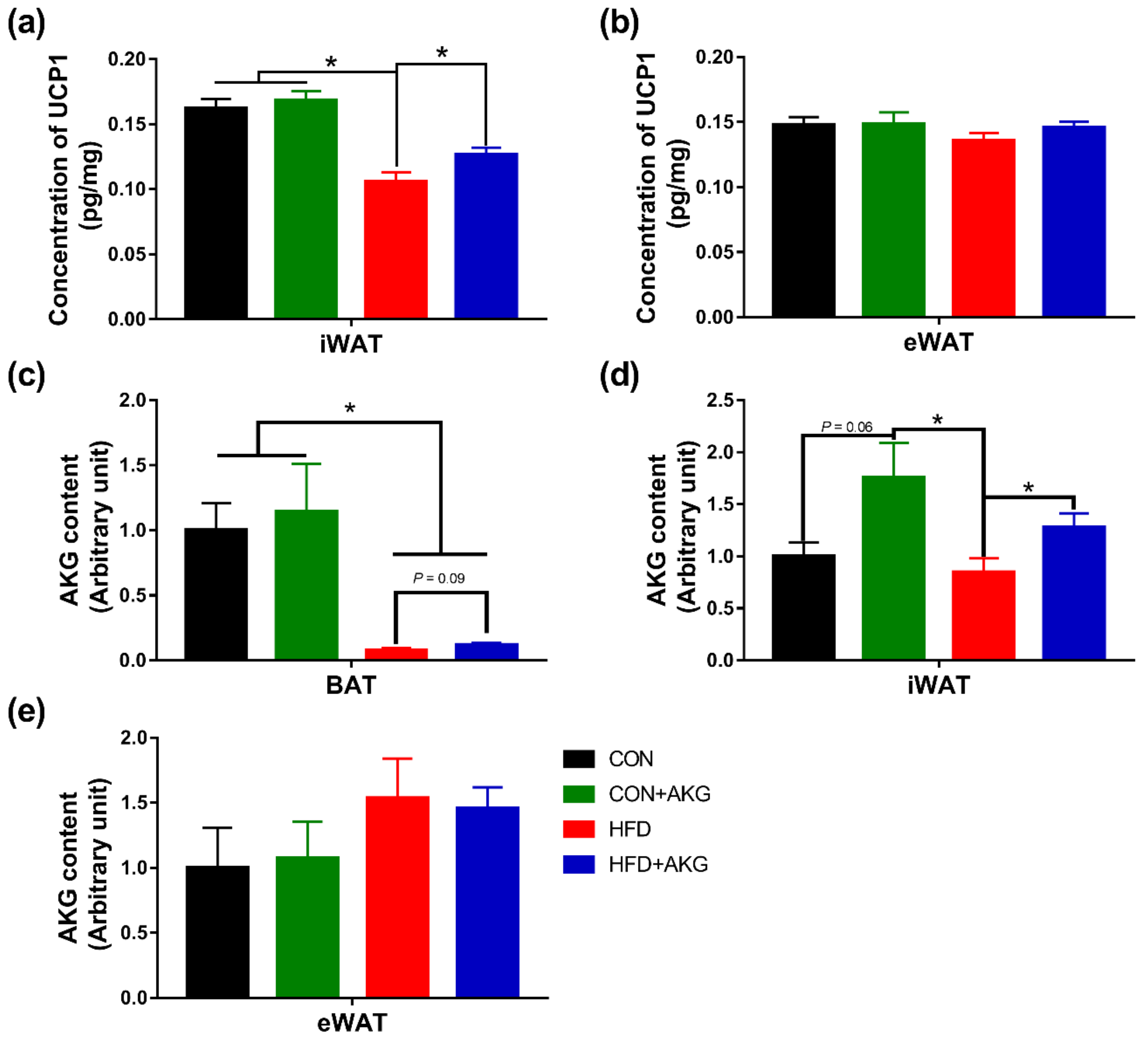

Supplementary Figure 7. UCP1 levels and AKG content in different adipose depots after cold exposure. (a) UCP-1 content in iWAT analyzed by q-PCR. (b) UCP-1 content in eWAT analyzed by q-PCR. (c-e) AKG content in BAT (c), iWAT (d) and eWAT (e). *$p < 0.05$ (n = 6, mean ± SEM).

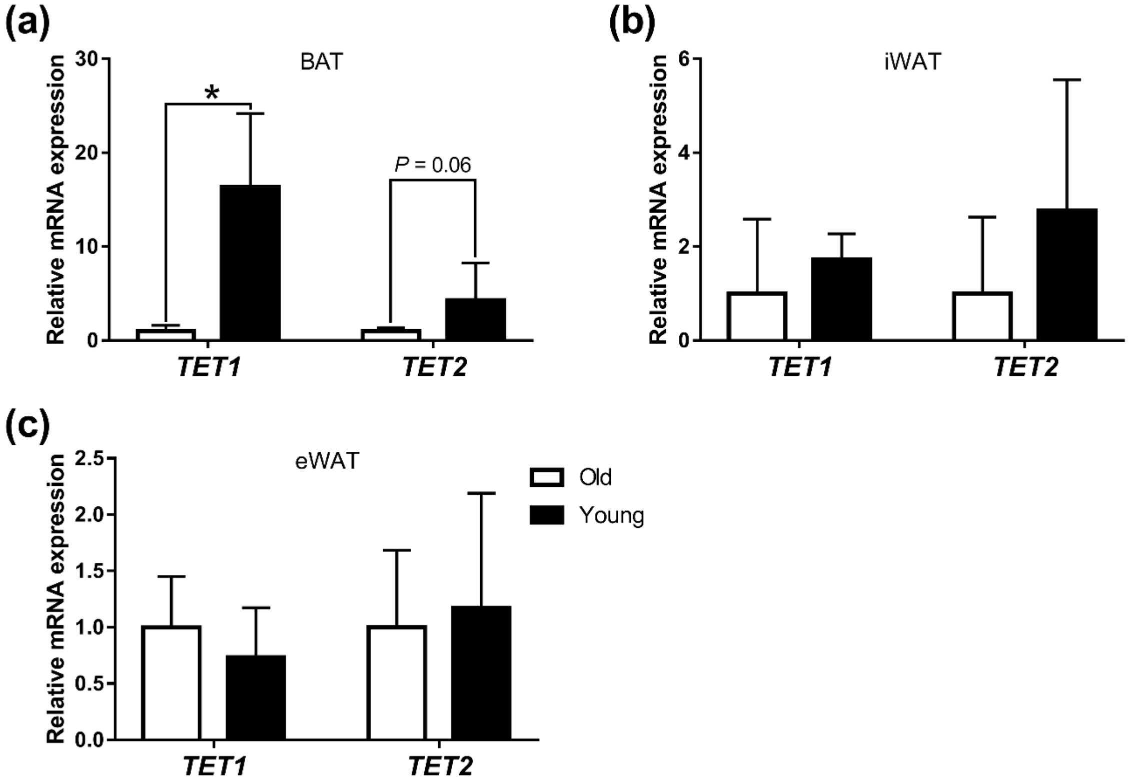

Supplementary Figure 8. Comparison of TET expression levels between young mice and middle-aged mice. (a) TET1 and TET2 mRNA expression in BAT analyzed by q-PCR. (b) TET1 and TET2 mRNA expression in iWAT analyzed by q-PCR. (c) TET1 and TET2 mRNA expression in eWAT analyzed by q-PCR. *$p < 0.05$ (n = 6, mean ± SEM).

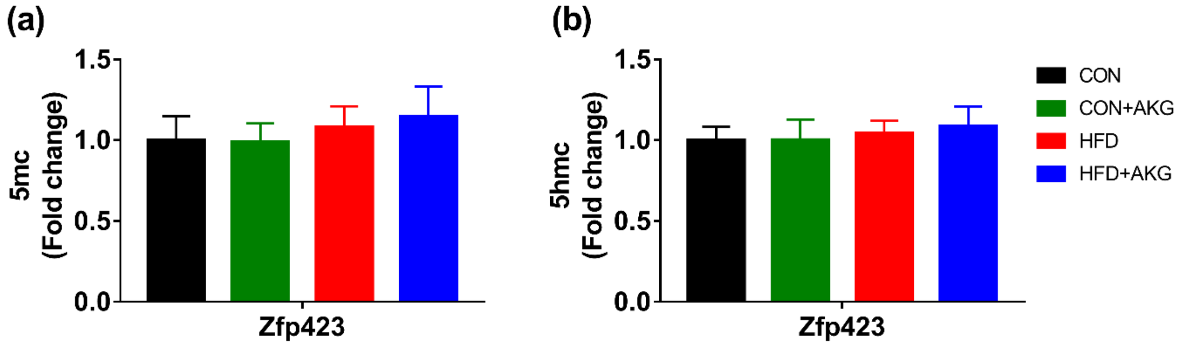

Supplementary Figure 9. DNA methylation of the *Zfp423* promoter after cold exposure. (a) Enrichment of 5mC at *Zfp423* promoter region relative to input normalized by the positive control in iWAT after cold exposure. (b) Enrichment of 5hmC at *Zfp423* promoter region relative to input normalized by the positive control in iWAT after cold exposure.
